# ‘*I just don't want to get bullied anymore, then I can lead a normal life*’; Insights into life as an obese adolescent and their views on obesity treatment

**DOI:** 10.1111/hex.12385

**Published:** 2015-07-17

**Authors:** Lindsey J. Reece, Paul Bissell, Robert J. Copeland

**Affiliations:** ^1^ Centre for Sport and Exercise ScienceSheffield Hallam UniversitySheffieldUK; ^2^ Professor School of Health and Related ResearchUniversity of SheffieldSheffieldUK; ^3^ National Centre for Sport and Exercise Medicine NCSEMSheffieldUK

**Keywords:** adolescent, bullying, emotional, obesity, treatment

## Abstract

**Background:**

Adolescent obesity is a complex condition involving social, emotional, behavioural and cultural issues.

**Design:**

One‐to‐one interviews and small focus groups with overweight and obese young people were conducted. Qualitative research is an appropriate method to explore the complexity of this issue.

**Setting and participants:**

Overweight and obese adolescent's attending a community weight management intervention in South Yorkshire.

**Main variables studied:**

Interviews aimed to explore the experiences of obese adolescents and their perspectives towards obesity treatment.

**Results:**

Adolescent's provided detailed accounts of their perspectives on weight gain, alluding to disordered patterns of eating and overeating, reported as being triggered by social and emotional factors, and in particular, bullying. Avoidance of bullying and a desire to integrate socially with peers were key drivers to seek treatment. Young people reported what they should do to lose weight, yet responsibility for successful weight loss and lifestyle change was repeatedly attributed to the treatment received, as opposed to viewing this as a combination of self‐motivation coupled with support provided by friends and family.

**Conclusion:**

Weight loss programmes need to consider the complex experience of obese young people in their design, focusing on how to implement long‐term lifestyle changes.

## Introduction

Adolescents in the United Kingdom (UK) have experienced the least improvement in health status of any age group in the last 50 years.^1^ A contributing factor could be the growing prevalence of overweight and obesity that continues to present a major public health and quality of life challenge for health‐care providers, policymakers, and adolescents themselves. Many studies have confirmed the significant physical impact on health in the short‐ and long‐term,^2^ particularly in a world which places increasing emphasis on body size as a marker of both self‐discipline and inner worth.^3^

Overweight and obese adolescents also experience a range of emotional consequences including low self‐esteem and self‐worth,^4^ difficulties in forming and sustaining peer relationships^5^ and poor educational outcomes.^6^ Bullying and victimization are also commonly reported as they seek to cope, particularly those who experience severe or morbid obesity.^7^ Young people who are overweight or obese in the UK are currently advised to attend family focused, lifestyle programmes that combine dietary modification, physical activity and psychological support although the quality and accessibility of obesity treatment is considered ‘patchy’ across different UK regions.^8^, ^9^ Evidence strongly suggests that these programmes have been ineffective in facilitating sustained weight loss amongst children and young people who are overweight or obese^10^ with poor psychosocial functioning identified in some studies as a potential barrier to living healthy happy lives.^11^, ^12^ The term psychosocial functioning can include a variety of meanings. When discussing overweight and obesity in relation to young people, psychosocial functioning relates to factors such as self‐esteem, self‐identity, perceptions of physical appearance, athletic skills and social functioning, all of which have been associated with a lower quality of life.^13^, ^14^ A range of studies show that poor family functioning, poor communication, poor behavioural control and high levels of conflict are associated with higher obesity levels.^15^ Additionally, adolescents living with obesity can experience a heterogeneous range of problems reinforcing their obesity, including poor family food practices, patterns of physical inactivity and sedentary behaviours, insufficient boundary setting around habits at home such as the use of technology and behaviours at mealtimes, whilst more complex issues around psychological well‐being and sexual or physical abuse also exist.^7^

Rapid changes during adolescence often cause anxiety that can confound existing feelings associated with living with obesity potentially influencing a referral to obesity services.^16^ Adolescence is thought to present a critical point for the successful implementation of obesity treatments^17^ such as physical activity and healthy eating programmes combined with behavioural change, to establish sustainable healthy lifestyle practices.^18^ Furthermore, the modification of the obesogenic family environment that focuses on broader determinants, not just the individual, is required.^19^

The family has been identified as one of the primary sites for socialization, laying the foundations of a young person's lifestyle habits, such as the establishment of healthy food practices.^20^ A key predictor of a child's weight status is driven by parental weight status.^21^ Parental role modelling helps foster an emotionally positive atmosphere,^7^, ^22^ and there has been considerable emphasis on the impact of familial habits fostering connectedness amongst peer relationships.^23^ Parents are frequently identified as the primary agents for fostering change,^24^ yet tensions can arise with teenagers seeking autonomy.^7^ Many parents do not recognize (or deny) that obesity may be an issue for their child, and when they do, their role in obesity treatment remains unclear.^19^ The mechanisms and processes through which parents perceive, influence and manage adolescent weight status were explored.^25^ It was found parents views of their own weight and the social and moral norms of labelling an adolescent overweight were crucial for service development. It is suggested that parents fail to acknowledge obesity in their children^26^ and the effectiveness of public health interventions aiming to tackle obesity are questioned, pointing to the need to involve parents within intervention programmes.

The complexity of issues contributing to child and adolescent obesity is not mirrored in current health policy relating to obesity, particularly in the English context. The national policy, ‘Healthy Lives, Healthy People: A Call to Action on Obesity in England’, views ‘*Overweight and obesity as a direct consequence of eating and drinking more calories and using up too few. We need to be honest with ourselves and recognise that we need to make some changes to control our weight’*. This is an example of the growing emphasis placed on personal responsibility in the context of epidemiological and sociological evidence of the complexity of health problems such as obesity.^3^ The use of this language can evoke feelings of blame and attributions of weakness from others and self,^27^ which has the potential to encourage individuals to enter potentially self‐defeating cycles of negative health behaviours. There are of course alternative ways of viewing the obesity epidemic as the Foresight whole systems map articulates.^28^ Foresight^28^ presents obesity as a function of a complex multifactorial system, encompassing biology and behaviour, all of which operate within a cultural, social and physical environmental network.^28^ There is at best an uneasy tension in current UK obesity policy between an emphasis on personal responsibility, and those that privilege sociostructural factors.

Being overweight or obese is well known as a risk factor for experiencing bullying and peer victimization^29^ with a well‐recognized detrimental impact of this on adolescent wellbeing.^30^, ^31^ Early adolescent years have also been associated with a need to ‘fit in’.^32^ Yet, obesity transgresses social norms with the overweight individual perceived as morally reprehensible,^33^, ^34^ which also shapes the identities and experiences. Stereotyping is also common^34^ resulting in obese people being stigmatized and associated with undesirable behaviours such as lazy, greedy and gluttonous.^35^

Weight management interventions for children and young people are associated with high attrition and dropout rates.^10^ Success is mainly associated with weight loss outcomes, largely ignoring qualitative data from the young people themselves that has the potential to inform intervention content^34^, ^35^ and ameliorate dropout. Previous studies that have assessed stakeholder perspectives towards obesity treatment^36^, ^37^, ^38^ suggest that congruence is needed between stakeholders on the content and purpose of treatment, recommending a collaborative approach towards treatment.^37^ Treatments must focus on tailoring for individual traits^39^ and incorporate the home environment into treatment to facilitate the transfer of behaviours learnt through the treatment process, into their everyday lives.^37^ To do this, however, a detailed understanding of what life is like for overweight and obese young is needed. To our knowledge, very little is still known about the experiences of obese adolescents, with few studies focusing on adolescent's perceptions of treatment.^16^ The aim of this study was to explore the adolescent experience of living with obesity and their engagement with obesity treatments, in order to help inform the evidence based for developing programmes that can support young people to manage their weight in the long‐term.

## Method

### Sample and setting

With National Health Service (NHS), ethics approval, 12 participants aged 11 to 16‐year‐old (4 male 8 female) were recruited, using a purposive sampling strategy, through attendance at community weight management programmes in South Yorkshire United Kingdom. All weight management programmes involved in the recruitment for this study adhere to NICE guidance^9^ and were delivered by multidisciplinary teams in community venues. Delivery incorporated healthy eating advice, varying degrees of behaviour change, physical activity advice and/or supervised physical activity sessions. Sessions are delivered by qualified lifestyle advisors, clinicians and physical activity specialists and can host up to 20‐25 individuals in group‐based sessions. Although individual characteristics were not captured such as sociodemographic information or weight status, to be eligible to attend, all young people must be clinically overweight, above the 91st centile. For the purposes of anonymity, all identifiable names have been removed.

### Procedures

Once informed consent (and parental consent) had been obtained, the young people (*n* = 12) took part in a semi‐structured, audio‐recorded interview held with the first author (LR), either on their own (*n* = 4), or in a small group with 2‐3 other young people (*n* = 3). All interviews and focus groups (*n* = 12) were conducted at the time and venue of the weight management club with interviews lasting 15–35 mins. A broad interview schedule was developed by the authors containing 12 open questions with prompts to support further exploration if needed. The schedule was developed after a review of current literature and consultation with key stakeholders involved in obesity management. The flexible guide included questions that focused on the exploration of lived experiences of obesity, its physical and psychological impact and participant's experiences of engaging with community‐based obesity treatments.^37^ Adhering to this guide ensured the interviews were consistent yet the researcher could remain flexible in their approach to allow for the emerging accounts of the young people. The interviewer (LR) has significant experience of behaviour change counselling with young people and conducting qualitative research with overweight and obese populations, allowing them to build rapport, listen and engage well with this cohort. Recruitment was supported through strong links with community deliverers who helped promote the study on researcher's behalf.

### Data analysis

All interviews were recorded and transcribed verbatim with all names and identifying information removed to preserve anonymity. The transcribed data were analysed using the Framework method^40^ led by LR. The initial stage of analysis involved familiarization with the data set by LR, resulting in the development of a list of recurrent themes. Data were annotated and indexed according to the emerging themes, all conducted in an intricate manner. The data set was then mapped and interpreted as a whole. To avoid bias and ensure trustworthiness of the data analysis, consultation took place frequently between researchers (LR and PB) as well as a review by an independent researcher neutral to the study, a method observed in previous published research.^37^

## Results

Results from the participant interviews are presented here in themes that emerged through the analysis process.^40^

### Accounts about the determinants of obesity

Young people were asked to speak about themselves generally, about hobbies and interests to open the dialogue for the interview. Participants were asked to recall experiences of being overweight, with responses prompted, where appropriate, with questions about food, physical activity and lifestyle at home. All of the young people (*n* = 12) volunteered reasons for being overweight, focusing on behaviours under their control, in line with previous studies.^7^, ^16^ Some offered accounts that focused on the combination of physical activity and dietary components of their lifestyles, whilst others spoke of an association between their emotions and their food related practices.

It's not what I eat but the amount I eat. I'll have my tea and I'll go into the fridge and get a yogurt or something or a bag of crisps

[Boy aged 15 yrs]

Whilst young people initially referred to ‘simple’ reflections of an energy imbalance in their lifestyle, it became apparent as the conversation continued, that this often obscured a much more complex context. In particular, many participants’ accounts were indicative of a problematic or disordered relationship with food, with many reporting that they ate as a response to upsetting events or particular social or familial cues. For example, some described eating because they felt sad, upset, anxious or bored, with a recognition that this behaviour contributed to their weight gain. Frequent allusions were made to patterns of weight cycling; weight loss and weight regain (this was also usually associated with unhappiness). The context of the home environment was eluded to briefly; however, this requires further exploration.

I think one of the reasons why I probably, I comfort eat a lot and there's like stuff going on, well used to be stuff going on at home which kind of like used to upset me a lot and I used to comfort eat

[Girl aged 15 yrs]

One of the key findings was that young people discussed accounts of bullying and stigmatization alongside their experiences of being obese.

I've been bullied a lot, makes me feel sad and upset

[Girl aged 14 yrs]

The majority of young people (*n* = 7) talked about being bullied by their peers, with many enduring name‐calling and social torment.

I used to get bullied a lot, and then when I got bullied I didn't eat, which made me put on more weight

[Boy aged 14 yrs]

There were numerous references to the negative impact of bullying on moods and emotions, with many talking openly about feeling sad, anxious and upset when bullying occurred. This reinforced feelings of low self‐worth and low self‐esteem.

I used to get bullied because of my weight and I want to do something about it but I used to kind of like want because I was getting bullied, either wanting comfort food and that of like made it worse because it made me bigger, which made me get bullied

[Girl aged 13 yrs]

None of the young people discussed seeking support for bullying, nor reported that their bullying situation had improved over time. Most participants appeared to believe that if they lost weight and looked like their peers, they would not be bullied and would thus feel better.

I just don't want to get bullied anymore, then I can lead a normal life

[Girl aged 16 yrs]

These accounts of being bullied appeared to act as a motivator to change and attend services, driven by the belief that the bullying would stop (*n* = 3). This is compounded by a strong sense of wanting to ‘fit in’ and be ‘normal’, rather than health‐related concerns.

I want to play with friends, get more out of breath and be the same as everyone else

[Girl aged 13 yrs]

I just don't want to get bullied anymore, and then like I cannot get picked on and then I can just do a normal life without getting stared at or something

[Girl aged 13 yrs]

This drive to be socially accepted was not articulated in a desire to look a certain way but through a need to fit in, to be like their peers, to be involved with activities they were doing, all to cease feelings of being isolated and socially withdrawn.

In contrast to some other studies,^41^ the young people interviewed here recognized the negative impact of excess weight on their physical health (*n* = 8), with one individual even considering the impact on her future health.

I'm not exactly happy with my appearance, and also because of health issues. I don't want to die of something to do with my weight, and I want to live like a nice long life basically

[Girl aged 16 yrs]

So it doesn't affect your life when you're older, like with all health problems and that

[Boy 14 yrs]

### Experiences with obesity treatments

Despite the young people talking freely about wanting to change, the scale of the challenge and concerns about how to maintain change were perceived as daunting.

You want to lose as much weight as your can but it's like an on‐going process

[Girl aged 16 yrs]

Interviews highlighted a high level of uncertainty about how to change health practices, with this extending to a lack of awareness from their parents too. Given the important role parents play in supporting young people to change, this finding has implications and requires further exploration beyond the scope of this study.

You want to change and as much as people were being horrible, it's like, I didn't really know how to deal with [it] nor did my mum either

[Girl aged 13 yrs]

A strong consensus emerged that primary support for weight loss came from the facilitators and peers involved with the weight management programme, with all young people commending the support they received (*n* = 12). All reported receiving concise messages regarding lifestyle changes during treatment.

It were like activities and stuff, like trying to get you active, trying to change your food portions, swap like junk food for fruit and stuff

[Boy 15 yrs]

They [weight management programme] encouraged me to do more sports, but I liked joining [weight management programme] you get information to help you cut down what you eat and also portion sizes, and they do activities for you as well

[Boy 15 yrs]

The young people emphasized the importance of social support, embracing the opportunity to build peer relationships and valued these highly during the treatment programme (*n* = 8).

Everyone bonds, its like its new. It's weird but everyone becomes really close

[Girl aged 13 yrs]

One significant finding was that young people in this study consistently attributed successful weight loss to the professional support they had received during treatment. Some even described the experience as a significant event in their life (*n* = 6).

Yeah, but then because it [weight management programme] stops after a bit doesn't it, then I just like, fell back into what I was doing before, because it were only like, I can't remember how long it were but it were short and I just fell back into what I were doing before

[Boy 15 yrs]

Well before I started coming to [weight management programme] and everything I weren't too happy with the way I were, but then I came here and it were alright and I got better

[Girl aged 15 yrs]

It's all been good and it's all helped, they've taught us everything we need to know and then it's just like us going out and doing it for ourselves

[Boy 16 yrs]

The biggest challenge expressed by young people was the perceived and actual difficulty in maintaining lifestyle changes post‐treatment. As a result, continued support and follow‐up were specifically recommended by participants (*n* = 5). This is supported by previous research with overweight and obese children and adoles‐cents.^24^, ^37^

I think if you just leave it I think that when you fall by the wayside. I think you've got to keep coming on a regular basis so that you're, you know, you're thinking about it all the time

[Boy aged 14 yrs]

It's not short term, I mean you do your six weeks and then that's it but it takes more than six weeks, it's a lifetime thing isn't it

[Girl aged 15 yrs]

Young people also recognized the challenges with providing longer‐term support but believed that this was needed for successful weight loss.

### Awareness and beliefs around alterative treatment approaches

Several young people had a general awareness of alternative treatment options referring to stories shared by people they knew or from magazines and the media (*n* = 10). Medication and bariatric surgery were mentioned briefly (*n* = 6) yet participants displayed only limited awareness about what the treatments entailed. This perhaps reflects the widespread adoption of the notion of personal responsibility for weight status, the young people felt using these methods represented a form of ‘cheating’ and that they would prefer to do it (weight loss) for themselves (*n* = 3).

I wouldn't do it. If you're going to lose weight you might as well do it to yourself not for other people. It's just cheating yourself

[Girl aged 15 yrs]

Sometimes they [obesity medication] don't work so there's no point in taking them. I'd rather do the work for myself so be proud of myself than taking tablets

[Boy aged 15 yrs]

I think she's [mum] had to like to get to a point where it's like you can't go back yourself and people do sometimes need surgery

[Girl aged 14 yrs]

Discussing the availability of treatment, there was strong agreement that treatments tailored to this age group were limited (*n* = 6).

I don't think there is anything really for them to, you know, join into and, you know, there's no support there really at all

[Girl aged 15 yrs]

Not really. Not for like people our age, maybe older like Weight Watchers and stuff like that, but not for young people

[Girl aged 15 yrs]

Despite an awareness of potential other treatments, young people perceived a treatment gap targeting their age group, and further exploration is needed to capture their perspectives on more invasive treatment methods.

## Discussion

The aim of this study was to explore adolescent experiences of living with obesity and their engagement with obesity treatments. We have structured the discussion around a number of key issues that emerged from the findings.

### Perspectives on weight management

#### Individual attribution to weight gain

The young people in this study provided detailed personal accounts about being overweight and obese. Examples of disordered dietary patterns, such as overeating and eating as a response to emotions such as boredom and sadness, were reported as contributors to weight status, which were often described as being the outcome of social and emotional factors, in particular – the experience of bullying. This is in line with previous findings^42^, where comfort and stress, along with mental health issues, were described by young people as contributing factors to their obesity. One of the major findings of the study was an overwhelming attribution of self‐blame and responsibility for being overweight or obese amongst these young people to themselves, mirroring widely held perceptions of a stigmatizing social environment.^34^ Previous research has presented a picture of overweight and obese young people experiencing feelings of low self‐esteem, low self‐worth, loneliness and difficulty forming interpersonal relationships,^43^ which when reinforced with feelings of blame and guilt – as also noted here – seem only likely to exacerbate weight gain. Findings from this study also highlight the challenging nature of the lived experience of overweight and obese amongst young people and point to some of the long‐term challenges associated with treatment, such as limited long‐term support and failure to maintain weight loss post‐intervention.^8^, ^17^ What is clear from this study is that programmes must consider the complex, lived experience of obese young people and their families^25^ in their design as well as considering how best to support them in long‐term change. Focusing on building self‐esteem, developing coping strategies for bullying and providing support to manage the broad ‐context with which obese adolescent's live within, such as the home environment^25^ and the broader social implications,^34^ appear necessary to successfully support positive healthful lifestyle change.

#### External attribution of successful weight loss

In the interviews, the focus of attention from young people in relation to treatment was on the professional support received from the weight management programme. Detailed accounts of knowledge gained from attending programmes were easily offered, with well‐rehearsed lifestyle messages around physical activity and diet commonly heard. There was also a strong sense of awareness amongst the young people of what they ‘should’ do to lose weight, and as reported earlier attributions of self‐blame. However, responsibility for successful weight loss and lifestyle change was repeatedly attributed to the programme, as opposed to acknowledging their own contribution and/or support offered from friends and family. This potentially diminishes their engagement with the weight loss process and undermines the development of self‐worth and esteem. There was also little to suggest that these young people had clear strategies on how to implement new practices into everyday life, especially when returning to their immediate family and social environments. This suggests, either, a strong emphasis of current programmes is on the ‘what’ rather than the ‘how’ of behaviour change, or that the internalization of key messages had simply not occurred. The dependence on the treatment described here ‐supports this, potentially explaining why the maintenance of successful behaviour change is so challenging for these young people. It appears essential that treatment programmes consider the ‘how’ as well as the ‘what’ of treatment in future.

#### Emotional and social aspects of obesity

Young people frequently described obesity in emotional terms using words such as sadness, upset and boredom. The family environment and bullying were explicitly offered as reasons contributing to overeating, and food was used as a way of responding to, or managing these elements, often described as a source of comfort.^31^ This reinforces the complex nature of obesity for these young people and supports the need to integrate changes to the home environment within treatment programmes. Researchers^44^ found adolescents struggled to make healthy choices at home and perceived many barriers to achieving this. The creation and maintenance of a supportive home environment requires consideration at multiple levels including parenting, relationships with siblings and modelling of appropriate practices and behaviours.^44^

There is much evidence confirming that being overweight is a risk factor for bullying,^46^ having detrimental effects with respect to adolescent's wellbeing^13^ whilst also reinforcing a lack of social skill development, thus enhancing the probability of being bullied.^46^ Without doubt, the findings here highlight the significant impact of bullying on the life‐experiences of these young people, but also its power as a motivator for change. However for many, this had not yet crystallized into sustained action at the level of health behaviours. Notwithstanding this, it is clear that the complex relationship between emotional upset, bullying, family dysfunction and weight gain require further longitudinal, qualitative studies to examine this relationship further.^46^

#### Reasons to change

Feelings of dissatisfaction with body image and physical appearance, and negative experiences of being bullied were described as reasons for wanting to change, a finding also observed in a previous review.^41^ This drive to avoid bullying and be socially accepted amongst their peers had acted as a catalyst for seeking obesity treatment amongst the young people interviewed here (although as we point out above, the internalization of messages to change health behaviours was problematic). Once engaged in treatment, there was unequivocal agreement amongst the young people, that the experience of interacting with peers in a socially supportive context was enjoyable and conducive to losing weight. At one level, this reinforces looking at physical activity determinants^42^ and the importance of factors such as enjoyment and having fun as being crucial for success. It seems just as likely that this may have been one of the first times that these young people had been able to physically interact with their peers in a safe and supportive environment, and crucial one where they were not stigmatized or shamed for being overweight or obese.

#### Critical comments on longer‐term weight loss strategies

Engagement was often not the most difficult part of the change process. Instead, the real challenge appeared to be maintaining new behaviours, and integrating them into their broader social contexts, particularly where the family and social environment was not supportive. It was also commonly held view that the loss of professional weight management support would inhibit their ability to sustain new behaviours and also negatively shape their motivation and willingness to change.

It is acknowledged that the evidence base identifying the key factors which enable adolescents to implement and maintain positive healthy behaviours is limited^23^ and draws into question the effectiveness of strategies within existing weight management programmes. Treatment programmes may unintentionally foster dependence rather than creating autonomous individuals who have the ‘life‐skills’ and emotional control to manage their behaviours and interact with their environment to achieve a healthy weight. Acknowledging the complex ways in which the broader family and social environment shapes adolescents health practices seems critical in maintaining long‐term change, as a focus on individual characteristics and the mobilization of personal resilience seem unlikely to be sufficient when viewed within the context of an increasingly obesogenic environment.^44^

### Study limitations

There are a number of limitations to this study. Although the purposive sampling strategy used here was suitable, it is important to recognize it is a convenience. The sample was also recruited through those actively engaged with a weight management club, and therefore does not capture the views of young people who had completed support, had dropped out or had never engaged with seeking support. The framework analysis approach^40^ adopted is popular amongst health‐care researchers, yet due to the openness within the analysis, views of the researchers could influence the codes that were applied, subsequently influencing the emerging themes. This was minimized by two researchers conducting the review process and similar strategies have been applied elsewhere.^37^

## Summary

The young people involved in this study provided detailed and often moving accounts of their experiences of obesity. The young people described negative and difficult emotions associated with being overweight and obese. Much of these negative experiences were associated with feelings of low self‐esteem, feeling ashamed or stigmatized accompanied by difficulties in making friends – all of which represented a catalyst to promote change. Bullying was described as debilitating, associated with behaviours promoting weight gain and reinforcing low self‐worth and self‐esteem. Young people associated their weight gain with their own behaviours and emotions, yet perceived the treatment programme as the way in which they would resolve their weight problems. This contradiction in relation to responsibility not only reinforces the need for further research into the effective delivery of long‐term weight management strategies, but also questions the appropriateness of the content of weight management treatment programmes for this population group. In the light of these findings, weight management programmes must be tailored to the specific needs of young people and find ways of integrating key messages and practices within their domestic environment, as much as possible. Programmes must aim to tackle the root cause of an individual's obesity, identifying and understanding their drivers for change. Young people require support to develop a skillset that enhances their ability to cope and manage emotional choices and situations if they are to develop a healthy pattern of lifestyle behaviours – merely educating on energy balance alone could be counter‐productive.^34^ It is recommended that young people are involved in the design of future weight management programmes working closely with overweight and obese young people. Exploration into the relationship between bullying and obesity is needed along with the role of families and the home environment. Further research must assess and identify the active ingredients within treatment programmes, which can support young people to manage their weight in the long‐term.

## Funding statement

This article presents independent research funded by the National Institute for Health Research Collaboration for Leadership in Applied Health Research and Care for South Yorkshire (NIHR CLAHRC SY) a pilot which ended in 2013. Further details about the new NIHR CLAHRC Yorkshire and Humber can be found at HYPERLINK ‘http://www.clahrc-yh.nihr.ac.uk’ www.clahrc-yh.nihr.ac.uk. The views and opinions expressed are those of the authors, and not necessarily those of the NHS, the NIHR or the Department of Health.

CLAHRC SY would also like to acknowledge the participation and resources of our partner organizations. Further details can be found at www.clahrc-sy.nihr.ac.uk.

## Conflict of interest

No conflict of interest to declared.
